# The *de novo* design of a biocompatible and functional integral membrane protein using minimal sequence complexity

**DOI:** 10.1038/s41598-018-31964-8

**Published:** 2018-10-01

**Authors:** Christophe J. Lalaurie, Virginie Dufour, Anna Meletiou, Sarah Ratcliffe, Abigail Harland, Olivia Wilson, Chiratchaya Vamasiri, Deborah K. Shoemark, Christopher Williams, Christopher J. Arthur, Richard B. Sessions, Matthew P. Crump, J. L. Ross Anderson, Paul Curnow

**Affiliations:** 1 School of Biochemistry, University of Bristol, Bristol, UK; 2 School of Chemistry, University of Bristol, Bristol, UK; 3 BrisSynBio, Life Sciences Building, Tyndall Avenue, Bristol, UK

## Abstract

The *de novo* design of integral membrane proteins remains a major challenge in protein chemistry. Here, we describe the bottom-up design of a genetically-encoded synthetic membrane protein comprising only four amino acids (L, S, G and W) in the transmembrane domains. This artificial sequence, which we call REAMP for **r**ecombinantly **e**xpressed **a**rtificial **m**embrane **p**rotein, is a single chain of 133 residues arranged into four antiparallel membrane-spanning α-helices. REAMP was overexpressed in *Escherichia coli* and localized to the cytoplasmic membrane with the intended transmembrane topology. Recombinant REAMP could be extracted from the cell membrane in detergent micelles and was robust and stable *in vitro*, containing helical secondary structure consistent with the original design. Engineered mono- and bis-histidine residues in the membrane domain of REAMP were able to coordinate heme *in vitro*, in a manner reminiscent of natural *b*-type cytochromes. This binding shifted the electrochemical potential of the cofactor, producing a synthetic hemoprotein capable of nascent redox catalysis. These results show that a highly reduced set of amino acids is sufficient to mimic some key properties of natural proteins, and that cellular biosynthesis is a viable route for the production of minimal *de novo* membrane sequences.

## Introduction

Integral membrane proteins play vital roles in biological systems including solute transport, signal transduction, and energy generation. Despite recent progress in structural biology, for many such proteins the relationships between sequence, structure and function remain unclear. One powerful means of understanding these relationships at a fundamental level is to design membrane proteins from first principles (*de novo*).

Previous studies have described chemically-synthesised *de novo* sequences that oligomerize to become channels, transporters, and electron transfer proteins in synthetic membranes^1–4^. An alternative and less well-explored strategy is to biosynthesise genetically-encoded *de novo* membrane proteins using the natural cellular machinery. Cellular expression is of interest since it provides access to long and hydrophobic amino acid sequences that are not chemically accessible, enables directed evolution and library screening in cell cultures, and could allow the expressed protein to influence the membrane biology of a living cell. The potential of cell expression has now been clearly demonstrated by a series of biocompatible synthetic membrane proteins obtained from the redesign of a soluble *de novo* sequence^5^.

Using expressible *de novo* proteins to explore multiple facets of membrane protein biology such as biosynthesis, targeting, topology control, folding, assembly, and cofactor binding is also an attractive proposition. The most powerful insights are likely to come from simple minimalist sequences that offer a neutral design background. This is borne out by the extensive use of model transmembrane helices with limited sequence diversity to investigate membrane insertion^6–10^, topology^11–13^, folding^14–18^ and assembly^19–21^. A logical extension of this approach is to develop new minimal systems that are geared towards a deeper understanding of oligomeric and multipass transmembrane proteins. It has already been shown that bottom-up designs with very low sequence complexity can be tolerated by the cell^11,22,23^, but it is not yet clear whether these simplistic proteins can bridge the gap between being both resident in cellular membranes and compatible with the *in vitro* biophysical methods necessary for protein characterization. A reasonable design target for such a protein would be a tetrahelical bundle, which is a common motif in natural membrane proteins and can support various functions^2–4^.

Here, we present a new minimal *de novo* protein that can be successfully expressed into the cytoplasmic membrane of *E*. *coli* and subsequently purified for further study. This sequence, named REAMP to denote a **r**ecombinantly **e**xpressed **a**rtificial **m**embrane **p**rotein, uses only Leu, Ser, Gly and Trp to define four transmembrane helices. The expression and cellular localization of REAMP were investigated and conditions were identified that could support REAMP purification and characterization *in vitro*. This sequence was then rationally engineered for cofactor binding, producing a synthetic metalloprotein with measurable catalytic activity. Our findings establish REAMP as a novel biocompatible scaffold that can imitate some features of natural membrane proteins.

## Results

### Protein design

To guide and constrain the design process we turned to the SMR family of small membrane proteins. Because our intention was to design a four-helix bundle, members of this family were of interest since the SMR protomer contains four contiguous and hydrophobic TM helices connected by short extramembrane loops. We identified eight particular SMR proteins (accession numbers P23895, Q2FD83, P69937, P14319, P69926, B0R6K7, P69210, P69213) which are among those to have been characterised and so are validated as authentic gene products. We reasoned that this deliberately restricted dataset, featuring diverse and functionally distinct proteins from *E*. *coli*, *A*. *baumanii*, *S*. *oneidensis*, *S*. *aureus*, *M tuberculosis*, and *H*. *salinarum*, would be sufficient to capture the general character of the natural amino acid sequences while avoiding bias towards any specific conserved residues or motifs^24^. We did not wish to recreate any specific secondary, tertiary or quaternary structure of the SMRs, nor to emulate the observed hydrophobic burial of functionally-important charged residues, but only to generate a basic template for our design.

**Figure 1:**
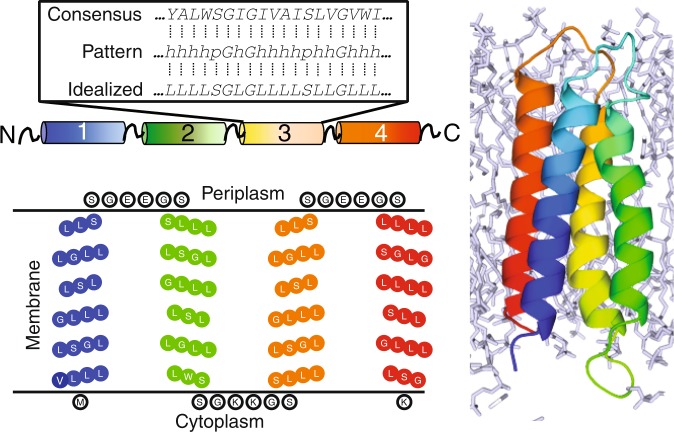
Amino acid sequence, predicted topology, and computational model of the *de novo* membrane protein REAMP. The boxed region illustrates how a consensus sequence from a small set of natural proteins was translated into an idealized sequence for each transmembrane helix.

Following a multiple sequence alignment, the consensus sequence of each transmembrane domain of the SMRs could be abstracted to the general pattern hhhhpGhGhhhhphhGhhhhp, where *h* = hydrophobic and *p* = polar (Supplementary Table 1 and Fig. 1, box). The length of this segment (21 residues) should be sufficient to span the hydrophobic core of a natural lipid bilayer. To translate this general pattern into an idealized *de novo* sequence, leucine (L) was introduced at every *h* position and serine (S) at every *p* position. We used these particular residues since L is the predominant amino acid in natural transmembrane proteins^25^ and is multifunctional, being able to engage in in protein-protein and protein-lipid interactions. S was selected as a small polar residue that is known to contribute to helix packing in membrane proteins^26^. Glycine (G) was retained at positions where it was relatively conserved in the consensus, given the particular role that it can play in promoting helix-helix interactions in the membrane^26–28^. Specific patterns and motifs known to occur in natural membrane proteins were otherwise deliberately avoided to minimize sequence complexity. Four helices each comprising the idealized minimal sequence LLLLSGLGLLLLSLLGLLLLS were linked by three flexible loops of sequence SSGXXGSS, where *X* was glutamate in loops one and three and lysine in loop two. This was to constrain the transmembrane topology of REAMP according to the ‘positive inside’ rule^11,29^. A single tryptophan (W) was introduced at residue 50, near the end of the second TM segment, to provide an optical signal, and for immunodetection a V5 antibody epitope was introduced at the C-terminus. Two different tags to allow purification by affinity chromatography were then introduced (see below). The sequences of all constructs are given in Supplementary Fig. S1.

Bioinformatic analysis predicted with reasonable confidence that REAMP would form four transmembrane α-helices and conform to the designed topology with N_in_/C_in_ orientation (Supplementary Table 2). Molecular dynamics simulations were used to further assess how REAMP would be accommodated within a generic lipid bilayer. In these simulations, REAMP rapidly approached a lower-energy structure relative to the starting conformation characterized by modest helical tilting and distortion that is most pronounced in helices 2 and 3 (Fig. 1). A video of this trajectory is included as Supporting Information. The transmembrane domains of REAMP are mildly amphipathic because of the serine residues (Fig. S2). During simulations the slightly polar faces of the helices remained facing the interior of the bundle, sequestered from the hydrophobic lipid phase.

### Protein expression and purification

**Figure 2:**
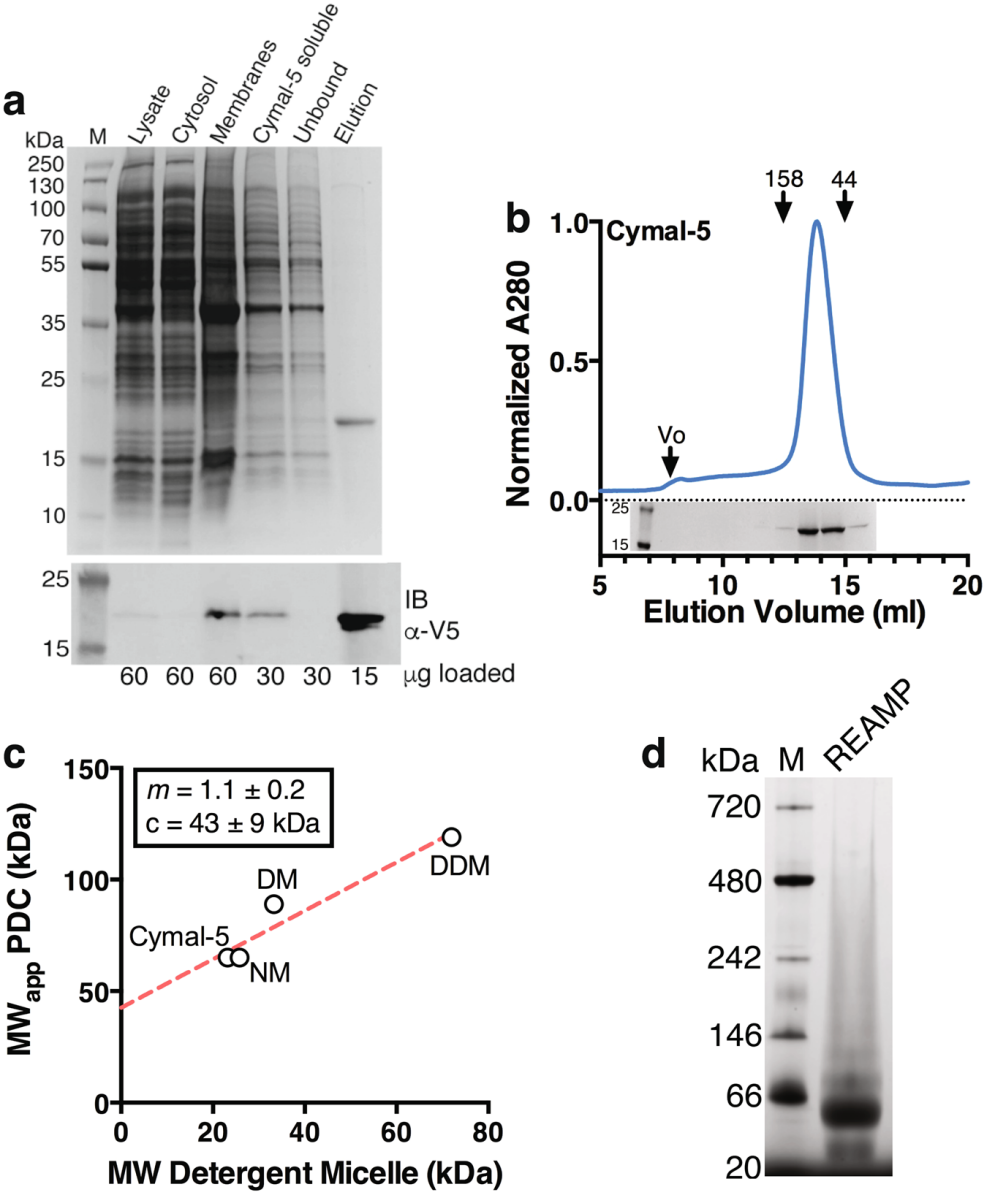
Affinity purification and characterization of REAMP. (**a**) Representative SDS-PAGE gel showing cell fractionation and purification of REAMP in Cymal-5. Immunodetection with anti-V5 (IB *α-V5*) confirms the protein identity and the success of the purification strategy. The uncropped blot is shown as Fig. S7. (**b**) Size exclusion chromatography shows that purified REAMP is homogenous and monodisperse in Cymal-5 with an apparent molecular weight of 65 kDa. SDS-PAGE analysis confirms that the peak is REAMP. The uncropped gel is presented as Fig. S8. (**c**) Purification of REAMP in other maltosides suggests that the protein contribution to the PDC is 43 ± 9 kDa, consistent with three protomers per micelle. Data are from SEC profiles shown in Fig. S9. (**d**) The PDC is apparently 60 kDa on blue native PAGE.

REAMP was synthesized as a synthetic gene and cloned into a recombinant vector for expression in *E*. *coli*. Western blotting against the V5 epitope was used to confirm this expression and to show that the expressed REAMP was localized to the cell membrane (Fig. 2a). We additionally confirmed the membrane localization with a REAMP-GFP fusion protein (Fig. S3). Cell membranes were isolated by centrifugation and REAMP was extracted as a protein-detergent-lipid complex. This allowed the purification of REAMP in a single step by affinity chromatography using either His_10_ tag or triple *Strep*-tagII affinity tags at the C-terminus. The results obtained with either of these two tags were indistinguishable.

A screen of the mild nonionic maltoside detergents showed that NM, DM, DDM and Cymal-5 could all support the purification of REAMP with yields of ~2 mg of purified protein per g total membrane protein. This is similar to yields obtained from the recombinant expression of natural membrane proteins. Interestingly, the REAMP-GFP fusion was produced at markedly higher levels of ~20 mg per g. REAMP was resolved as a single major species on SDS-PAGE, migrating close to the calculated theoretical weights of the His-tagged and triple *Strep*II-tagged constructs of 13.8 kDa and 17.8 kDa, respectively (Figs 2a, S4). In some instances purified REAMP ran as a doublet, suggesting multiple SDS binding isoforms (Figs 2a, S4). The identity of the purified protein was additionally confirmed by amino acid analysis and mass spectrometry (Figs S5, S6).

### Detergent compatibility and oligomeric state

We next assessed the oligomeric state, aggregation propensity and dispersity of purified REAMP with size-exclusion chromatography (SEC). REAMP was homogenous and monodisperse in DM and Cymal-5, but the elution profiles in DDM and NM were more heterogenous (Figs 2b; S9). Changes in the aggregation propensity in different detergent environments - even when those detergents are closely related - is a common characteristic of many membrane proteins. The elution volume of REAMP in Cymal-5 corresponded to a Stokes radius (*R*_*s*_) of 3.5 nm. This translates to an apparent molecular mass of 65 kDa versus protein standards. A lipid assay determined that no host cell lipid was co-purified with REAMP, and so this apparent mass represents the protein-detergent complex (PDC). This was unexpected given that the monomer mass of His_10_-tagged REAMP is 13.8 kDa, and that the Cymal-5 micelle would be expected to contribute only 20–25 kDa to the complex. We judged that a PDC mass of 65 could represent three REAMP protomers per micelle.

We further interrogated this apparent oligomerization of REAMP in detergent micelles. Blue-native PAGE agreed with SEC, showing a single band at 60 kDa (Fig. 2d). Particle sizing with solution dynamic light scattering gave the same *R*_*s*_ of 3.6 ± 0.2 nm (compared with 3.0 ± 0.1 nm for the empty micelle). Following Erickson^30^ we used gradient centrifugation to determine a sedimentation coefficient in Cymal-5 of 4.2 *S* relative to soluble protein standards (Fig. S10). Combining this with the Stokes radius according to Eq. 1 gave a molecular mass of 60 kDa for the PDC. A REAMP-GFP fusion protein also eluted from size exclusion at an apparent mass consistent with three protomers per micelle (Fig. S3). Within the maltoside series tested, the apparent mass of the lowest mass peak was linear with micelle size (Fig. 2c) and we applied the method described by Kunji^31^ to determine by extrapolation that the contribution of REAMP to the micelle was 43 ± 9 kDa. Finally, nanoelectrospray MS^32^ only gave a peak for the REAMP monomer even at low voltages, confirming that the apparent homotrimer readily dissociated and so was not inherently stable nor an unrecoverable aggregate (Fig. S6). Analytical ultracentrifugation was not possible because REAMP was not stable in C8E5. Collectively, these findings suggest that REAMP is adventitiously purified at three protomers per micelle, but that this does not represent a specific aggregation complex.

### Structure and topology

**Figure 3:**
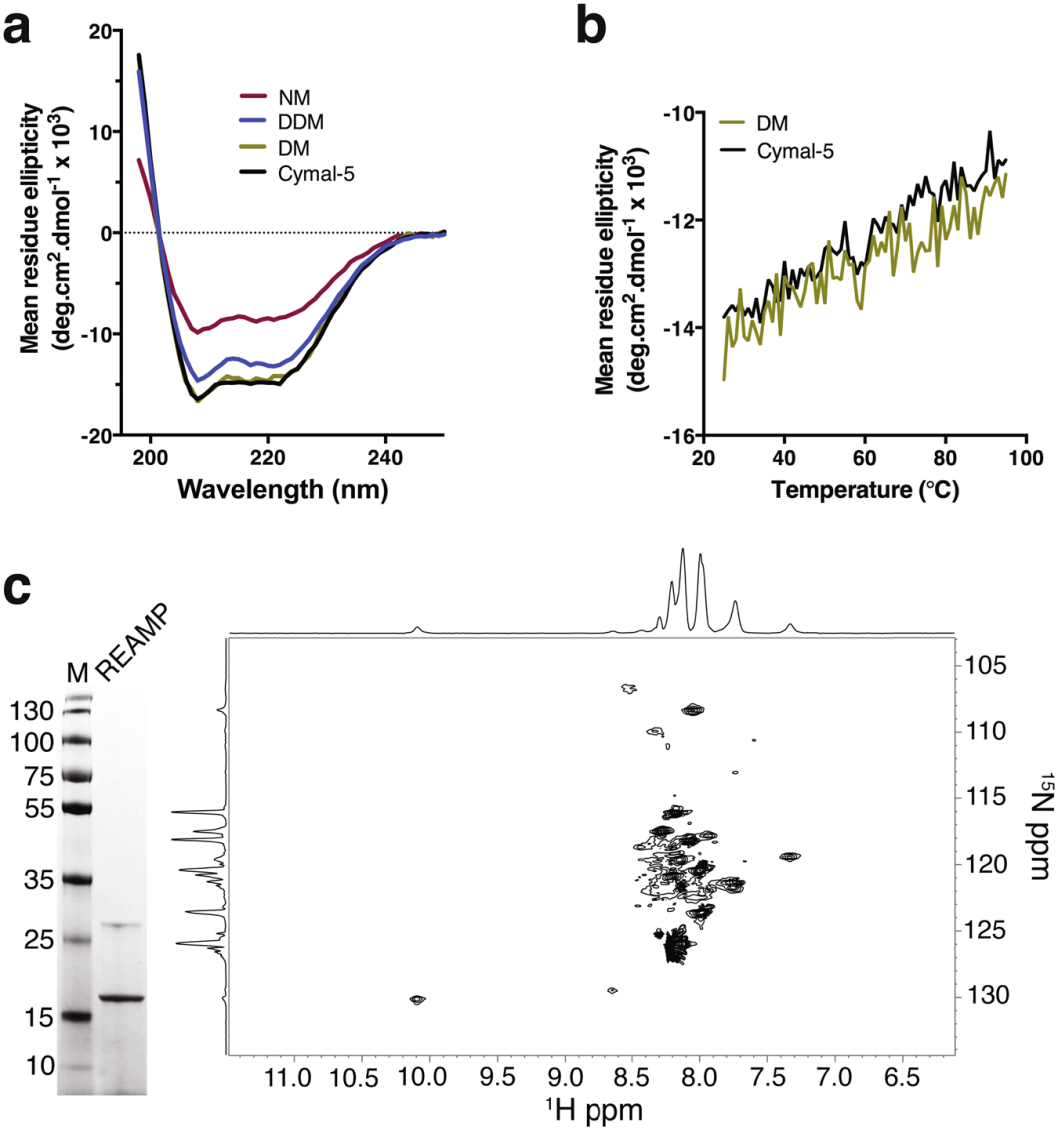
Secondary and tertiary structure of REAMP. (**a**) Circular Dichroism shows that DM and Cymal-5 support the highest degree of secondary structure. (**b**) Melting curves at 222 nm. (**c**) ^1^H-^15^N TROSY-HSQC spectrum of REAMP in Cymal-5 at 40 °C.

The structure and transmembrane topology of REAMP were characterized by circular dichroism (CD), NMR, and cysteine scanning mutagenesis. UV-CD showed that REAMP was clearly α-helical in all of the detergents tested, with strong negative deflections at 208 nm and 222 nm (Figs 3a, S11). Data recorded in DM and Cymal-5 were indistinguishable, and both the shape and intensity of the spectra suggested ~60% helix overall. Helicity was slightly reduced in DDM and markedly reduced in NM. CD was also used to probe the thermal stability of REAMP (Fig. 3b). The CD signal showed a linear decrease to ~80% of the starting signal at 95 °C, typical for both natural membrane proteins and other *de novo* membrane sequences because of the high energetic cost of unfolding an alpha-helix in a hydrophobic environment^4^.

The ^1^H-^15^N HSQC NMR spectrum of REAMP generally showed poor chemical shift dispersion and resolution, although a few well-dispersed peaks could be observed (such as the Trp indole proton at 10.1 ppm in the ^1^H dimension). This result most likely reflects a combination of the slow tumbling of the large PDC and the highly repetitive amino acid sequence, and may also indicate that the protein behaves as a dynamic molten globule. Although this requires further investigation beyond the scope of the current work, many *de novo* proteins are conformationally heterogeneous^33–36^ and this would be unsurprising since the REAMP design strategy did not dictate specific tertiary interactions. Engineering specific packing interactions into the minimal REAMP sequence should allow for improved structural definition in future design iterations.

**Figure 4:**
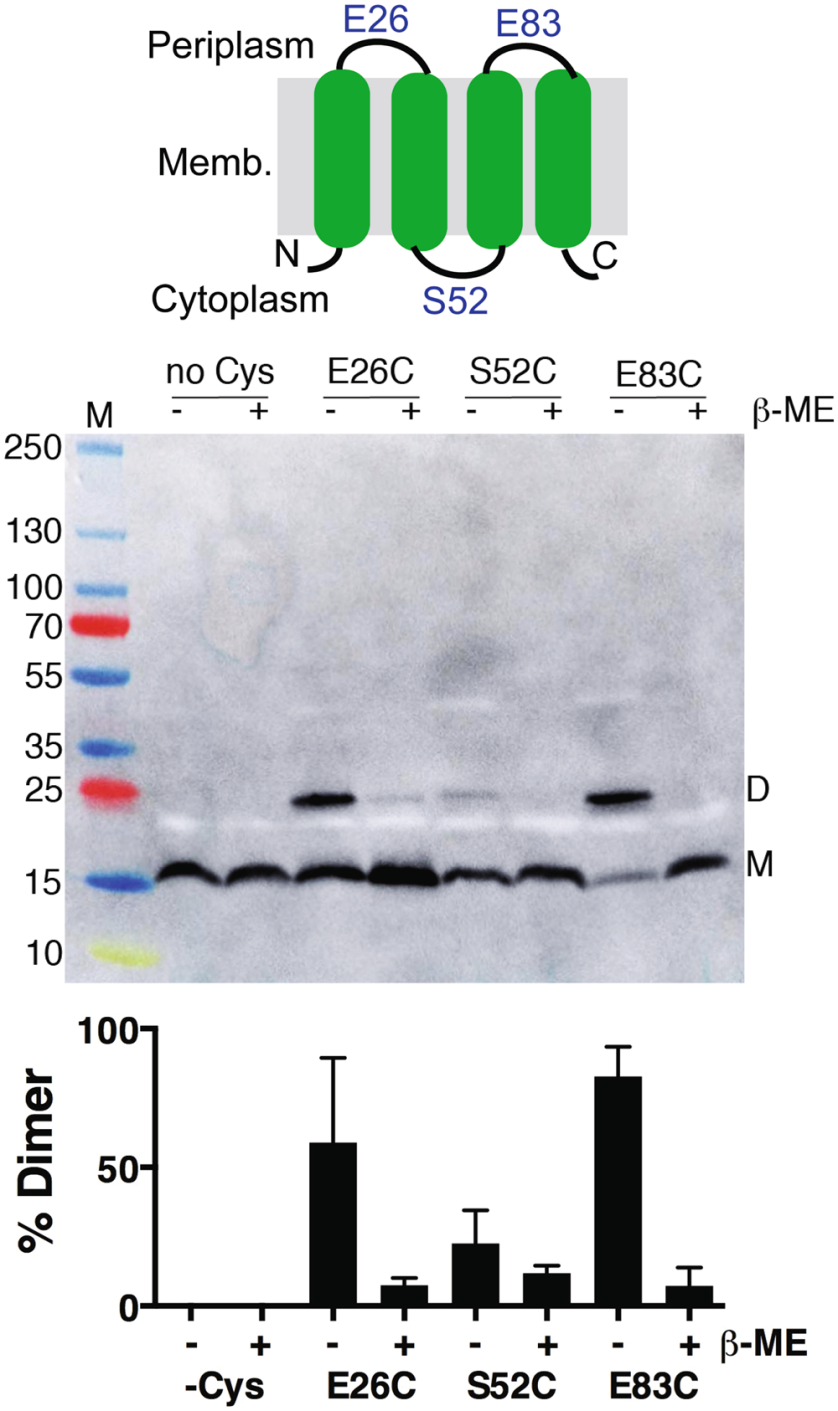
Transmembrane topology of REAMP. Introducing Cys residues in the periplasmic loops (E26C, E83C) results in apparent covalent dimer bands (*D*) that are abolished by the reducing reagent β-mercaptoethanol (β-ME). Top panel, representative western blot; bottom panel, percent dimer per lane (mean ± s.d., *n* = 3).

Cysteine accessibility experiments were used to probe the transmembrane topology of REAMP. Single Cys residues were introduced into the extramembrane loops to replace E26, S52, and E83. These experiments were carried out in a S35H/L94H/L22A/L75A mutant background (see below) because this variant gave a much stronger western blot signal than the original parent sequence but otherwise behaved identically. When cysteines were placed in the periplasmic loops at E23C or E83C an intense band was observed in western blots of crude cell lysate and cell membrane fractions that presumably corresponded to a REAMP dimer (Fig. 4). This band accounted for approximately 50% and 95% of the total protein for E26C and E83C respectively. The presumed dimer band was abolished by the reducing reagent β-mercaptoethanol (β-ME), suggesting that it arises from inter-protein disulphide bonds formed in the oxidizing environment of the periplasm. In contrast, introducing a cysteine into the intracellular loop (S52C) showed no such dimer band, consistent with this linker being sequestered in the reducing environment of the cytoplasm. It thus appears that the topology of REAMP conforms to design, whereby the first and third loops are periplasmic and the second loop is intracellular.

### Cofactor binding

Many natural proteins exploit a cofactor to achieve their biological function. One of the most prominent cofactors in membrane biochemistry is heme, which is responsible for electron transfer during photosynthesis and oxidative phosphorylation. In terms of *de novo* design, engineering heme binding is of interest because of the functional diversity of heme metalloenzymes and because a well-defined binding site and tertiary structure are not necessarily required; indeed, heme binding can drive the ordering of *de novo* molten globules^34^. Designed and re-designed sequences that bind to heme and related porphyrins have thus previously been studied in micelles and model lipid membranes^2,4,37–40^. The most common axial ligand for heme binding in both natural proteins and artificial proteins is histidine in either a mono-His or bis-His configuration^41–43^.

The computational model of REAMP (Fig. 1) was used to identify amino acids facing the protein interior that were candidates for mutation to His. This selection was refined by applying the design criteria that the residue should be buried into the membrane by ~10 Å to allow partitioning of the bulk of the porphyrin ring. For bis-His coordination, two residues were selected that were diametrically opposed across the 4-helix bundle. To help relieve steric clashes between the porphyrin ring and inward-facing leucine residues a crude binding site was ‘hollowed out’ by replacing L22 and L75 with alanine.

This process resulted in one mono-His and two bis-His variants of REAMP in a L22A/L75A background: REAMP^S35H^, REAMP^S35H/S90H^ and REAMP^S35H/L94H^, where S35 is on helix 2 and S90 and L94 are on helix 4. Each of these variants was purified as an apoprotein in Cymal-5 as described above for REAMP (Fig. S12). Purification yields were similar to the REAMP parent or even slightly better in the case of REAMP^S35H/L94H^, which also gave a stronger signal on western blots. We thus used REAMP^S35H/L94H^ as our bis-His construct.

**Figure 5:**
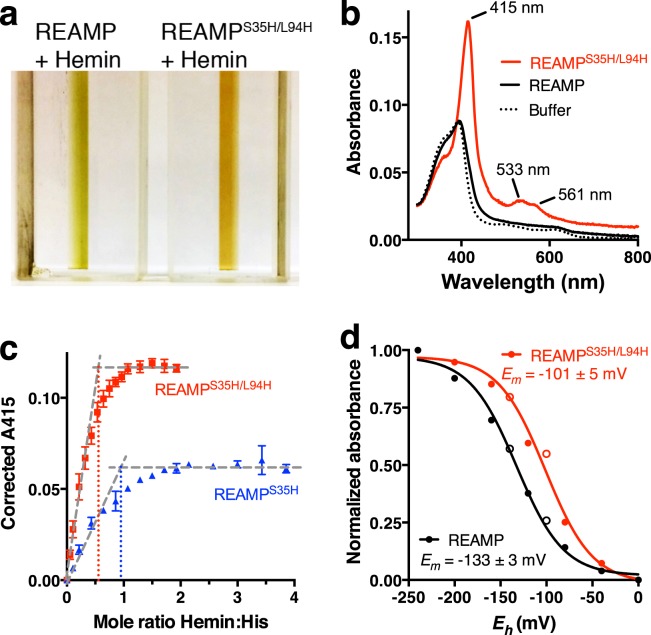
Heme coordination. (**a**) Adding heme to a bis-Histidine variant of REAMP causes a colour change from green to red indicative of cofactor binding. (**b**) Representative UV/Vis spectroscopy showing spectral changes characteristic of heme binding to REAMP^S35H/L94H^. (**c**) Both mono-His and bis-His variants exhibited tight binding. Data are mean ± s.d, *n* = 3. (**d**) Bis-His coordination shifts the heme redox potential. Values are from fitting the reductive sweep (closed symbols) to the Nernst equation for a single electron reduction.

Heme binding to these variants was assessed by UV/Vis absorbance spectroscopy (Fig. 5). A bulk detergent concentration of 0.1x CMC was sufficient to keep the protein stable while eliminating the background signal that arises from heme partitioning into empty micelles. Under these low-detergent conditions, introducing heme (as hemin) to REAMP gave data similar to a buffer-only control, showing a broad absorbance band with a modest peak at 394 nm (Fig. 5b). These samples remained brownish-green to the eye indicating that the heme remained in a relatively aqueous environment (Fig. 5a, left). In contrast, adding heme to REAMP^S35H/L94H^ produced a visible red colour (Fig. 5a, right) and gave spectra consistent with hydrophobic burial and bis-His heme coordination (Fig. 5b). The Soret (B) band was red-shifted to 415 nm and other spectral features emerged in the Q-region between 500–700 nm (Fig. 5b). When these samples were reduced either electrochemically or with sodium dithionite the Soret peak was shifted to 427 nm and the Q-bands were substantially sharpened (Figs S13, S15). There were subtle but observable differences between the heme spectra of mono-His and bis-His mutants (Fig. S13). The absorbance of the mono-His spectrum was less intense, with a shoulder toward shorter wavelengths. We were unable to study the effects of heme binding by protein NMR because of significant visible aggregation when the apoproteins were concentrated in dilute detergents.

All histidine variants showed tight binding of heme once the nonspecific background signal from the REAMP control had been subtracted. The binding stoichiometry was as expected based on design, being 1:1 heme:His and 0.5:1 heme:His for mono-His and bis-His coordination, respectively (Fig. 5c). The apparent equilibrium dissociation constants (*K*_*d*_) were 2.9 ± 0.2 μM for REAMP^S35H^ and 2.2 ± 0.1 μM for REAMP^S35H/L94H^. The equivalent value for REAMP, albeit with a much smaller signal, was 5.6 ± 0.9 μM. Kinetic analysis showed that cofactor binding to the bis-His variant is rapid and is characterized by having both a faster on-rate and a slower off-rate than the REAMP control (Fig. S14). Values for *K*_*d*_ derived from kinetic data (*k*_*off*_ /*k*_*on*_) were similar to those from equilibrium measurements, being 1.7 ± 0.4 μM for REAMP^S35H/L94H^ and 4.5 ± 0.4 μM for REAMP.

Redox potentiometry was used to measure the midpoint equilibrium potential (*E*_*m*_) of both REAMP^S35H/L94H^ and REAMP in the presence of heme (Figs 5d, S15). For REAMP, *E*_*m*_ was −133 ± 3 mV *vs* standard hydrogen electrode. This is very close to previous values obtained for heme partitioned into detergent micelles^38^, where the reduction potential is increased over aqueous solvents because of the energetic cost of burying ferric heme within the hydrophobic micelle interior. For REAMP^S35H/L94H^ the *E*_*m*_ was shifted by +32 mV to −101 ± 5 mV. The most likely cause of this shift is coordination by at least one histidine and increased desolvation.

### Peroxidase activity

**Figure 6:**
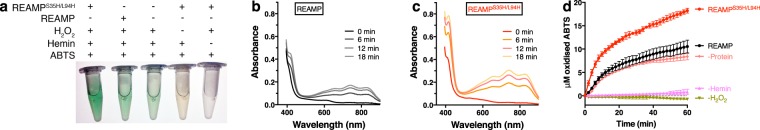
Redox catalysis by the REAMP^S35H/L94H^ hemoprotein. (**a**) Photograph showing ABTS oxidation to a dark green product under the conditions used. (**b**,**c**) Representative absorbance scans showing the increased activity of the hemoprotein *versus* REAMP. (**d**) The evolution of oxidized ABTS by the REAMP^S35H/L94H^ hemoprotein can be distinguished from background controls. These controls are REAMP + Heme (*REAMP*), heme only in the absence of any protein (*-Protein*), REAMP^S35H/L94H^ apoprotein (*-Hemin*) and REAMP^S35H/L94H^ hemoprotein with peroxide omitted (*-H*_2_*O*_2_). Data are mean ± s.d., *n* = 3.

A number of previous studies have shown that *de novo* metalloproteins can take part in electron transfer reactions^34,44–46^. The REAMP^S35H/L94H^ hemoprotein was used in a classical peroxidase assay in which the reduction of H_2_O_2_ was coupled to the oxidation of ABTS. The results of this assay suggested that the REAMP^S35H/L94H^ hemoprotein was indeed active in electron transfer, albeit to a very limited degree (Fig. 6). The REAMP^S35H/L94H^ hemoprotein slowly evolved a modest but distinguishable signal above control samples that corresponded to about 7 reactions per hemoprotein. This suggested that the catalytic activity was likely to be accompanied by rapid poisoning of the hemoprotein.

## Discussion

The engineering and design of α-helical and β-barrel membrane proteins is now receiving increased attention^47,48^ but remains an important problem that is relevant to both basic and applied science. One potential barrier to progress is that membrane proteins are notoriously difficult to produce recombinantly, because sequences that are so strongly biased towards hydrophobic amino acids are liable to aggregate. However, the results presented here show that the minimal *de novo* sequence REAMP is expressible, can be successfully processed by the cell, and is stable after purification. The implications of this are discussed below.

Although it is well-attested that fusion proteins can support the expression of synthetic transmembrane segments, it is less clear that this extends to standalone *de novo* sequences and examples are relatively scarce. The available evidence is that redesigned and *de novo* proteins comprised of a limited selection of hydrophobic amino acids (mainly L, I and A) can generally be expressed by the cell^11,22,23,40^ and localized to the plasma membrane^11,22,23^. Goparaju and colleagues^40^ used a fusion protein strategy to deliberately express a minimal *de novo* protein into inclusion bodies, so the intrinsic localization of that sequence is unknown. It thus appears that austere *de novo* membrane sequences of low complexity are broadly compatible with cellular expression. In the case of REAMP, this sequence appears to be rather malleable and can be mutated with little impact on protein expression; indeed some mutants are produced at higher yield than the parent construct. It will be interesting to interrogate random and rational sequence libraries of REAMP to determine which amino acid substitutions, in which positions, can be tolerated by the cell. Understanding the design parameters for the successful biogenesis of REAMP could help to identify generic principles that underpin the recombinant production of integral membrane proteins. Any such insight might support the rational engineering of natural proteins to improve expression yields.

The results here also show that REAMP could be readily purified and characterised in the maltoside family of detergents (Figs 3, 5 and 6). Why was this protein so robust *in vitro*? Presumably the simplicity and small size of REAMP mean that there are few hydrophobic regions exposed from the detergent micelle. Additionally, REAMP is not evolved to suit a particular membrane environment or to make stabilizing interactions that cannot be satisfactorily mimicked by a detergent. REAMP purifies in an aggregation state of three proteins per micelle for unknown reasons, and it should be possible to engineer this oligomerization by changing the protein sequence. REAMP may thus represent a simple model for understanding the specific and non-specific association of membrane proteins.

The design strategy for this first iteration of REAMP was deliberately simplistic and only tried to define the topology of secondary structure elements. NMR analysis of REAMP is consistent with a dynamic molten globule (Fig. 3), although this remains to be confirmed. Several investigators have found that molten globule states are good starting points for protein design^33,36,49^. The structural definition of REAMP could likely be improved by introducing specific packing motifs and other general features found in natural membrane proteins^26–28^. Similar strategies could also be employed to optimise and manipulate cofactor binding with the ultimate goal of assembling a catalytic hemoprotein *in vivo*. Such a sequence could be the basis for a synthetic bioenergetic protein capable of biological electron transfer, or of acting as a novel enzyme at the cell membrane.

The prevalence and importance of metalloenzymes in biology has led to extensive efforts to incorporate metal cofactors into artificial proteins^46,50,51^. Of closest relevance here are those studies that have focused upon helical membrane domains. For example, the transmembrane region of the natural membrane protein Glycophorin A was subtly redesigned to generate synthetic peptide dimers that bind heme in micelles via bis-His coordination. The apparent *K*_*d*_ for heme binding was 0.5 μM, slightly lower than the 2.2 μM reported here for REAMP derivatives^38^. The redox potential of this hemoprotein was −128 mV, so it does not show the same shift to higher voltages that we observe for REAMP^S35H/L94H^. More recently, the antimicrobial peptide VK22 was the inspiration for a membrane peptide dimer called HETPRO that exploited histidine ligation to bind two hemes with *K*_*d*_^*app*^ of 2.9 μM^37^. Both the redesigned Glycophorin A and HETPRO were also active in peroxidase assays, analogous to the activity shown here for the REAMP hemoprotein^37,38^. As well as peptide dimers, four-helix bundles have also been designed that can incorporate multiple copies of both natural and artificial porphyrins. In one example, the lipophilic peptide PRIME spontaneously assembles into a tetramer in detergent micelles and tightly binds the non-natural iron diphenylporphyrin at each of two distinct bis-His sites^2^. Goparaju and colleagues also used histidine to coordinate different porphyrins at individual sites within a tetrahelical peptide bundle, and extended this to include a highly minimal sequence that can be recovered from cellular inclusion bodies^40^. This latter hemoprotein showed a substantial positive shift in redox potential, probably +100 mV above that seen here for REAMP derivatives, and it was possible to achieve light-activated electron transfer between the bound cofactors^40^. This work drew at least partly upon the prior design of amphiphilic *de novo* proteins, in which a hydrophobic section can be appended to a hydrophilic domain, and which can be engineered to include a porphyrin binding region directly inspired by the sequences of natural *b*-type cytochromes^4^. These constructs use histidine ligation to coordinate a variety of different porphyrins in the lipophilic segment, and heme binding in particular can be very tight with *K*_*d*_ being 50 nM in the most favourable cases^4^. Collectively, these examples suggest that there is scope to improve the heme binding affinity of REAMP, and to explore whether REAMP derivatives can bind to other metalloporphyrins. Additionally, it should be possible to rationally modulate the redox potential of the REAMP hemoprotein and to engage multiple cofactors with a single protomer for intra- and interprotein electron transfer.

We thus show here that a deliberately naïve, minimal artificial sequence is successfully recognized by the cell machinery and can broadly imitate aspects of natural membrane proteins *in vivo* and *in vitro*. Future iterations of REAMP should be useful in probing the fundamental mechanisms of membrane protein biogenesis and assembly, and for developing functional *de novo* membrane proteins that interact with the cell. Overall, our results provide further evidence for the general feasibility of membrane protein design.

## Materials and Methods

### Protein expression

A synthetic gene corresponding to the REAMP sequence with a C-terminal V5 epitope was obtained from DNA2.0 (now ATUM, Inc.). The synthetic gene fragment was digested with the restriction enzymes NcoI and XhoI to allow cohesive end ligation into a modified version of the expression vector pET28c. This strategy placed the expression of REAMP under the T7 promoter, allowed selection by kanamycin and introduced a His_10_ tag at the C-terminal. The C-terminal His-tag was replaced by a triple *Strep*-tag II sequence^52^ by cohesive end ligation of a synthetic DNA fragment after XhoI digestion. The same strategy was used to introduce the superfolder variant of GFP (sfGFP) at the C-terminal, with the codon for sfGFP L141 changed from CTC to CTG in order to remove an internal XhoI site. All constructs were confirmed by sequencing.

REAMP was expressed in *E*. *coli* strain BL21-AI (Invitrogen). Primary cultures of 100 ml were grown from a single bacterial colony overnight at 250 rpm, 37 °C in LB media. For protein purification, 10 ml of this primary culture was removed into each of three secondary 1 L cultures in 2.5 L baffled flasks. We found empirically that more mature cultures (A600 > 1) gave a higher final protein yield because of the overall greater cell mass. Induction was for two hours with 0.1 mM IPTG and 0.1% arabinose. Analytical cell lysates were prepared as required from small-scale cultures using the BugBuster reagent (Novagen).

### Protein purification

The secondary cultures were harvested by centrifugation at 4000 × g for 15 min. The supernatant was discarded and the pellet resuspended in ~200 ml phosphate buffered saline (PBS). This suspension was briefly homogenized by hand before cell lysis in a continuous flow cell disruptor (Constant Systems Ltd) at 25 kpsi. The lysate was centrifuged at 10,000 × g for 10 min to remove unbroken cells and other debris. The cell membranes were then isolated from this clarified lysate by ultracentrifugation at 170,000 × g for 1 h. The supernatant was discarded, and the membrane pellet was resuspended in Buffer A (50 mM sodium phosphate buffer, pH 7.4, 150 mM NaCl, 5% glycerol). This membrane suspension was subject to at least 10 passes in a hand-held homogenizer before being treated with a solubilizing detergent. This was one of either 5-cyclohexylpentyl-β-D-maltoside (Cymal-5), nonyl-β-D-maltoside (NM), decyl-β-D-maltoside (DM) or dodecyl-β-D-maltoside (DDM) obtained from Anatrace at the highest purity and used at >10x critical micelle concentration. After incubation with gentle agitation for 1 h at room temperature, insoluble material was removed by ultracentrifugation as above in order to isolate soluble protein-detergent-lipid complexes (PDLCs).

For purification by immobilized metal affinity chromatography, a 1 ml Ni-NTA ‘HisTrap’ column (GE Healthcare) was equilibrated in Buffer B (Buffer A with the detergent of choice at ≥2x critical micelle concentration) plus 20 mM imidazole. Soluble PDLCs, also in 20 mM imidazole, were applied to the equilibrated column at a flow rate of 1 ml/min. The loaded column was then washed with 40 column volumes of Buffer B with 75 mM imidazole. Purified REAMP as a protein-detergent complex (PDC) was recovered by elution in Buffer B with 0.5 M imidazole at a flow rate of 0.2 ml/min. Imidazole was immediately removed using a PD-10 desalting column (GE Healthcare) equilibrated in Buffer B. A centrifugal concentrator with either 30, 50 or 100 kDa molecular weight cut-off, depending on detergent, was used to bring purified REAMP to 2 mg/ml typically in 0.3–0.5 ml volume. Small aliquots (20 μl) were snap-frozen in liquid N_2_ and stored at −80 °C.

For purification using the triple *Strep*-tag II, membranes were solubilised in Buffer S (50 mM Tris, pH 8.0, 150 mM NaCl, 5% Glycerol) plus 2.4% Cymal-5. After removing insoluble material by centrifugation as above, soluble protein was loaded onto a 1 ml *Strep*-tactin column (IBA) at a flow rate of 1 ml/min. After washing with 20 column volumes of Buffer S plus 0.24% Cymal-5, REAMP-strep was eluted with 8 column volumes of Buffer S plus 0.24% Cymal-5 and 2.5 mM *D*-Desthiobiotin. The purified protein was concentrated with a 50 kDa MWCO cut-off centrifugal concentrator.

### Protein characterization

The total protein concentration in cell and purification fractions was determined with a detergent-compatible Lowry assay (DC Protein Assay, BioRad). Co-purified phospholipid was determined with a commercial assay kit (Sigma-Aldrich). Amino acid analysis was performed by Alta Biosciences. The concentration of purified REAMP was determined from the absorbance at 280 nm using a calculated extinction coefficient of 5500/M/cm and a theoretical molecular weight of 13,757 Da (http://web.expasy.org/protparam/). For REAMP-Strep3 these values are 22,000/M/cm and 17, 830 Da, respectively.

SDS-PAGE analysis used commercial precast 10%, 12% or 4–20% acrylamide Tris-Glycine gels (NuSep or SERVA). Western blots were analysed with ImageJ^53^.

The following proteins were used as standards: Ferritin (molecular weight 440 kDa, Stokes radius 6.1 nm), Aldolase (158 kDa, 4.8 nm, sedimentation coefficient 7.3 *S*), conalbumin (75 kDa, 3.6 nm, 5.1 *S*), ovalbumin (44 kDa, 3.1 nm, 3.5 *S*). Size exclusion chromatography (SEC) was performed on an Äkta system using a Superdex S200 10/300 column (GE Healthcare). The void volume of this column was determined with Blue Dextran. For preparative size-exclusion of REAMP and mutants, the column was equilibrated with 1.5 column volumes of Buffer B before <1 ml of sample was injected at a flow rate of 0.5 ml/min. Size-exclusion data were analysed as described by Kunji *et al*.^31^.

Circular dichroism was performed on a Jasco J-1500 instrument at 0.2–0.5 mg/ml protein in a 0.1 cm pathlength cell. The purified protein was diluted into 5 mM sodium phosphate, pH 7.4, 135 mM NaF with 0.24% Cymal-5. Buffer backgrounds were collected and subtracted from each experiment. Wavelengths at which the high-tension (HT) voltage exceeded 700 V were excluded. Data were analysed with the SELCON3, CONTIN and K2D programs available at the Dichroweb server (http://dichroweb.cryst.bbk.ac.uk/html/home.shtml)^54^.

Protein sedimentation analysis was performed in a continuous 5–20% sucrose gradient with 0.24% Cymal-5 to maintain protein solubility. An 80 μl sample consisting of REAMP at 1.4 mg/ml and the calibration standards Aldolase, Conalbumin and Ovalbumin at 1 mg/ml was loaded onto a 2 ml gradient and ultracentrifuged in a Beckman TLS-55 swing-out rotor for 6 h at 118,000 × g. Molecular weight (*M*) in kDa was determined as described by Erickson^30^ using Eq. 1:

1 $${M}=4.205({S}\cdot {{R}}_{{s}})$$

where *S* is sedimentation coefficient (Svedberg units) obtained from sucrose gradient sedimentation and *R*_*s*_ is Stokes’ radius (nm) from size-exclusion and solution dynamic light scattering.

^15^N-labelled samples of REAMP were prepared from cells grown in supplemented minimal media as described^55^. Purified protein was concentrated to 5 mg/ml in Buffer A plus 0.24% Cymal-5 and 10% D_2_O, before being transferred to 1.7 mm NMR tubes. All NMR experiments were performed on a Brucker Avance III HD 700 MHz NMR instrument equipped with a 1.7 mm TCI microcryoprobe at 40 °C. Excitation sculpting 1D ^1^H spectra with water suppression (zgesgp) were acquired using 1024 scans per experiment, an interscan delay of 1 s and a spectral width of 14 ppm with 32 K complex points to check the quality of samples. ^1^H-^15^N BEST-TROSY spectra (b_trosyf3gpph.2) were acquired with a spectral width of 14 ppm in ^1^H and 33 ppm in ^15^N with a relaxation delay of 0.2 s. The spectra were processed using NMRPipe^56^ and analysed using CcpNmr Analysis version 2.4.1^57^.

Mass spectrometry was carried out essentially as described^32^ in 0.2 M ammonium acetate, 0.24% Cymal-5.

### Molecular Dynamics

A molecular model of REAMP was made as follows. Four α-helices were built and positioned with Chimera, decorated and packed using SCWRL4, and the loops built with Modeller^58,59^. Hydrogen atoms were added consistent with pH 7 and the model was embedded in a POPC lipid bilayer in a box of SPC waters containing 0.15 M NaCl. The system was parameterized with the OPLS/AA and Berger-lipid forcefields and energy minimized for 5000 steps prior to molecular dynamics simulations. Simulations were performed for 200 ns at 310 K using periodic boundary conditions and PME long-range electrostatics with GROMACS-4.6.7. Presentation figures were prepared with PyMol.

### Heme binding

Heme binding assays were carried out in Buffer A. Hemin was freshly diluted to 150 μM and introduced in small aliquots to 1 ml of 1.4 μM protein in a 1 cm pathlength cuvette. Cymal-5 was maintained at 0.012% and data were collected after a 10 s equilibration. Kinetic data were recorded in identical buffers using a stopped-flow instrument (Applied Photophysics) in absorbance mode.

For redox potentiometry, the protein was diluted to 50 μM in Buffer A plus 0.012% Cymal-5 before Hemin was added to a 2-fold excess. Potentiometry was performed as described^60^ and the data were fit to the Nernst equation for a one-electron reduction (Eq. 2):

2 $${\rm{A}}{\rm{b}}{\rm{s}}{\rm{o}}{\rm{r}}{\rm{b}}{\rm{a}}{\rm{n}}{\rm{c}}{\rm{e}}={{\rm{A}}{\rm{b}}{\rm{s}}}_{min}+({{\rm{A}}{\rm{b}}{\rm{s}}}_{max}/({10}^{{{\rm{E}}}_{{\rm{m}}}{\textstyle  \mbox{-} }{{\rm{E}}}_{{\rm{h}}}/59})+1)$$

where Abs_min_ and Abs_max_ are the minimum and maximum observed absorbance signals respectively, *E*_*m*_ is the midpoint potential and *E*_*h*_ is the ambient redox potential with respect to the standard hydrogen electrode. Incubation times of 30 min were required to reach equilibrium at each voltage, and over longer timescales this led to hysteresis in the reverse (oxidizing) sweep of REAMP^S35H/L94H^. This may arise from oxidative damage to the detergent micelle which destabilizes the membrane protein.

Electron transfer assays were performed in Buffer A plus 0.012% Cymal-5 at 1.4 μM protein, 1 μM hemin, 0.0025% H_2_O_2_, 1 mM ABTS at 25 °C. The extinction coefficient of ABTS at 420 nm was taken as 36,000 /M /cm.

## Electronic supplementary material

Supplementary information

Supplementary video

## Data Availability

All of the raw data underpinning this work is available from the authors on request.
