# Supplementary material for: The de novo design of a biocompatible and functional integral membrane protein using minimal sequence complexity
Source: Sci Rep. 2018 Oct 1;8:14564. doi: 10.1038/s41598-018-31964-8 (PMC6167376; doi:10.1038/s41598-018-31964-8)
Supplement: Supplementary file 1 — Supplementary information [file 41598_2018_31964_MOESM1_ESM.pdf]

SUPPLEMENTARY INFORMATION

The *de novo* design of a biocompatible and functional integral membrane protein using minimal sequence complexity

Authors: Christophe J. Lalaurie[1], Virginie Dufour[1,3], Anna Meletiou[1], Sarah Ratcliffe[1], Abigail Harland[1], Olivia Wilson[1], Chiratchaya Vamasiri[1], Deborah K. Shoemark[1,3], Christopher Williams[2,3], Christopher J. Arthur[2], Richard B. Sessions[1,3], Matthew P. Crump[2,3], J.L Ross Anderson[1,3] and Paul Curnow[1,3]*

Affiliations: [1]School of Biochemistry and [2]School of Chemistry, University of Bristol, UK. [3]BrisSynBio, Life Sciences Building, Tyndall Avenue, Bristol, UK.

*Corresponding author. Email: p.curnow@bristol.ac.uk

List of contents

Supplementary Table 1. Consensus from multiple sequence alignment of SMR proteins used to derive the abstracted general pattern as shown.

Fig. S1. DNA and protein sequence of the His_{10}-tagged, (*Strep*-tag II)$_3$–tagged and GFP fusion derivatives of the synthetic membrane protein REAMP.

Supplementary Table 2. Bioinformatic analyses predict that REAMP will be a multipass membrane protein comprising four transmembrane α-helices with N_{in}/C_{in} topology

Fig. S2. Helical wheel representation showing mild amphipathicity of each helix of REAMP.

Fig. S3. Purification of REAMP-sfGFP-His_{10} (REAMP-GFP).

Fig. S4. SDS-PAGE gel of purified REAMP-His_{10}.

Fig. S5. Amino acid analysis of purified REAMP-Strep3, and comparison to theoretical protein composition.

Fig. S6. Mass spectrometry of REAMP-Strep3.

Fig. S7. Uncropped western blot of REAMP-Strep3 purification.

Fig. S8. Uncropped Coomassie-stained SDS-PAGE gel analyzing fractions from the size-exclusion chromatography of REAMP-Strep3.

Fig. S9. Purification of REAMP in four different maltoside detergents as described in the text.

Fig. S10. Sucrose density gradient ultracentrifugation of REAMP.

Fig. S11. Circular dichroism spectroscopy of REAMP-Strep in Cymal-5 confirms the expected α-helical structure.

Fig. S12. Coomassie-stained SDS-PAGE gels showing the His-tag purification of bis-His and mono-His variants as shown.

Fig. S13. Spectra of oxidized and dithionite-reduced heme co-ordinated by the mono-His and bis-His mutants of REAMP.

Fig. S14. Kinetics of heme binding.

Fig. S15. Redox potentiometry.

SUPPLEMENTARY TABLE 1

Supplementary Table 1. The consensus sequence from a multiple sequence alignment of the transmembrane helices of a small set of SMR proteins. This was used to derive the abstracted general pattern as shown. Details are given in the main text.

TM Helix	Consensus sequence
1	MPYIYLAIAIAAEVVGTSALK
2	LIPSVGTLVGYGASFYLLSLT
3	YALWSGIGIVAISLVGVWILF
4	LDLMKIVGLALIVAGVVILNL
Pattern	*hhhhpGhGhhhhphhGhhhhp*

Red = nonpolar; **Green = uncharged polar**; **Blue = charged polar**

SUPPLEMENTARY FIGURE S1

>REAMP-V5-His$_{10}$
ATGGTTCTGTTGCTGCTGTCTGGTCTGGGTTTGTTGCTGTTGTCTTTGCTGGGTTTGCTGTT
GCTGAGCAGCGGTGAGGAGGGTAGCAGCCTGTTGCTGTTATCCGGTCTGGGTCTGCTGCTGT
TGAGCCTGCTGGGCTTACTGCTGTGGAGCAGCGGTAAAAAGGGCAGCAGCCTGCTTCTGCTG
TCAGGTCTGGGCCTCCTGCTGCTGAGCCTGTTGGGCCTGCTGCTTCTGTCCAGCGGTGAAGA
AGGCTCCCTGCTGCTGCTCAGCGGCCTGGGCTTGCTGCTGCTGTCGCTGCTGGGCCTGCTCC
TGCTGAGCGGTAAACCGATCCCGAACCCGCTGCTGGGTCTGGACAGCACCTCACTCGAGCAT
CATCATCATCACCACCACCACCACCACTGA

>REAMP-V5-His10_translated
MVLLLLSGLGLLLLSLLGLLLLSSGEEGSSLLLLSGLGLLLLSLLGLLLWSSGKKGSSLL
LLSGLGLLLLSLLGLLLLSSGEEGSLLLLSGLGLLLLSLLGLLLLSGKPIPNPLLGLDST
SLEHHHHHHHHHH-

>REAMP-V5-Strep3
ATGGTTCTGTTGCTGCTGTCTGGTCTGGGTTTGTTGCTGTTGTCTTTGCTGGGTTTGCTGTT
GCTGAGCAGCGGTGAGGAGGGTAGCAGCCTGTTGCTGTTATCCGGTCTGGGTCTGCTGCTGT
TGAGCCTGCTGGGCTTACTGCTGTGGAGCAGCGGTAAAAAGGGCAGCAGCCTGCTTCTGCTG
TCAGGTCTGGGCCTCCTGCTGCTGAGCCTGTTGGGCCTGCTGCTTCTGTCCAGCGGTGAAGA
AGGCTCCCTGCTGCTGCTCAGCGGCCTGGGCTTGCTGCTGCTGTCGCTGCTGGGCCTGCTCC
TGCTGAGCGGTAAACCGATCCCGAACCCGCTGCTGGGTCTGGACAGCACCTCACTCGAGGGT
GGTGGGTCTGGTGGTGGGAGCGGTGGAGGCAGCTGGTCGCATCCGCAGTTTGAGAAGGGCGG
CGGATCAGGCGGCGGATCCGGCGGTGGCTCGTGGTCCCATCCGCAATTCGAGAAGGGTGGCG
GCAGTGGTGGCGGCTCTGGCGGTGGGTCGTGGAGCCACCCACAGTTCGAAAAGTGA

>REAMP-V5-Strep3_translated
MVLLLLSGLGLLLLSLLGLLLLSSGEEGSSLLLLSGLGLLLLSLLGLLLWSSGKKGSSLL
LLSGLGLLLLSLLGLLLLSSGEEGSLLLLSGLGLLLLSLLGLLLLSGKPIPNPLLGLDST
SLEGGGSGGGSGGGSWSHPQFEKGGGSGGGSGGGSWSHPQFEKGGGSGGGSGGGSWSHPQ
FEK-

>REAMP-V5-GFP
ATGGTTCTGTTGCTGCTGTCTGGTCTGGGTTTGTTGCTGTTGTCTTTGCTGGGTTTGCTGTT
GCTGAGCAGCGGTGAGGAGGGTAGCAGCCTGTTGCTGTTATCCGGTCTGGGTCTGCTGCTGT
TGAGCCTGCTGGGCTTACTGCTGTGGAGCAGCGGTAAAAAGGGCAGCAGCCTGCTTCTGCTG
TCAGGTCTGGGCCTCCTGCTGCTGAGCCTGTTGGGCCTGCTGCTTCTGTCCAGCGGTGAAGA
AGGCTCCCTGCTGCTGCTCAGCGGCCTGGGCTTGCTGCTGCTGTCGCTGCTGGGCCTGCTCC
TGCTGAGCGGTAAACCGATCCCGAACCCGCTGCTGGGTCTGGACAGCACCTCACTCGAGATG
AGTAAAGGAGAAGAACTTTTCACTGGAGTTGTCCCAATTCTTGTTGAATTAGATGGTGATGT
TAATGGGCACAAATTTTCTGTCCGTGGAGAGGGTGAAGGTGATGCTACAAACGGAAAACTCA
CCCTTAAATTTATTTGCACTACTGGAAAACTACCTGTTCCGTGGCCAACACTTGTCACTACT
CTGACCTATGGTGTTCAATGCTTTTCCCGTTATCCGGATCACATGAAACGGCATGACTTTTT
CAAGAGTGCCATGCCCGAAGGTTATGTACAGGAACGCACTATATCTTTCAAAGATGACGGGA
CCTACAAGACGCGTGCTGAAGTCAAGTTTGAAGGTGATACCCTTGTTAATCGTATCGAGTTA
AAGGGTATTGATTTTAAAGAAGATGGAAACATTCTTGGACACAAACTGGAGTACAACTTTAA
CTCACACAATGTATACATCACGGCAGACAAACAAAAGAATGGAATCAAAGCTAACTTCAAAA
TTCGCCACAACGTTGAAGATGGTTCCGTTCAACTAGCAGACCATTATCAACAAAATACTCCA
ATTGGCGATGGCCCTGTCCTTTTACCAGACAACCATTACCTGTCGACACAATCTGTCCTTTC
GAAAGATCCCAACGAAAAGCGTGACCACATGGTCCTTCTTGAGTTTGTAACTGCTGCTGGGA
TTACACATGGCATGGATGAGCTCTACAAACTCGAGCATCATCATCATCACCACCACCACCAC
CACTGA

```
>REAMP-V5-GFP_translated

MVLLLLSGLGLLLLSLLGLLLLSSGEEGSSLLLLSGLGLLLLSLLGLLLWSSGKKGSSLLLL
SGLGLLLLSLLGLLLLSSGEEGSLLLLSGLGLLLLSLLGLLLLSGKPIPNPLLGLDSTSLEM
SKGEELFTGVVPILVELDGDVNGHKFSVRGEGEGDATNGKLTLKFICTTGKLPVPWPTLVTT
LTYGVQCFSRYPDHMKRHDFFKSAMPEGYVQERTISFKDDGTYKTRAEVKFEGDTLVNRIEL
KGIDFKEDGNILGHKLEYNFNSHNVYITADKQKNGIKANFKIRHNVEDGSVQLADHYQQNTP
IGDGPVLLPDNHYLSTQSVLSKDPNEKRDHMVLLEFVTAAGITHGMDELYKLEHHHHHHHHH
H
```

Figure S1. DNA and protein sequences of the REAMP constructs used in this study.

SUPPLEMENTARY TABLE 2

Method	Nterm	TM1	TM2	TM3	TM4	Cterm
TMPred	in	3-22	31-50	59-78	86-105	in
TMHMM[1]	in	2-24	28-50	57-79	84-107	in
HMMTOP[2]	in	6-24	31-50	59-78	85-104	in
Philius[3]	in	5-23	31-50	59-78	84-104	in
SCAMPI2[4]	in	2-22	31-51	58-78	85-105	in

Supplementary Table 2: Bioinformatic analyses predict that REAMP will be a multipass membrane protein comprising four transmembrane α-helices with N_{in}/C_{in} topology. TMPred was accessed via http://embnet.vital-it.ch/software/TMPRED_form.html; TMHMM, http://www.cbs.dtu.dk/services/TMHMM/; HMMTOP, http://www.enzim.hu/hmmtop/; Philius, via the TOPCONS[5] server http://topcons.net; SCAMPI2, http://scampi.bioinfo.se.

SUPPLEMENTARY FIGURE S2

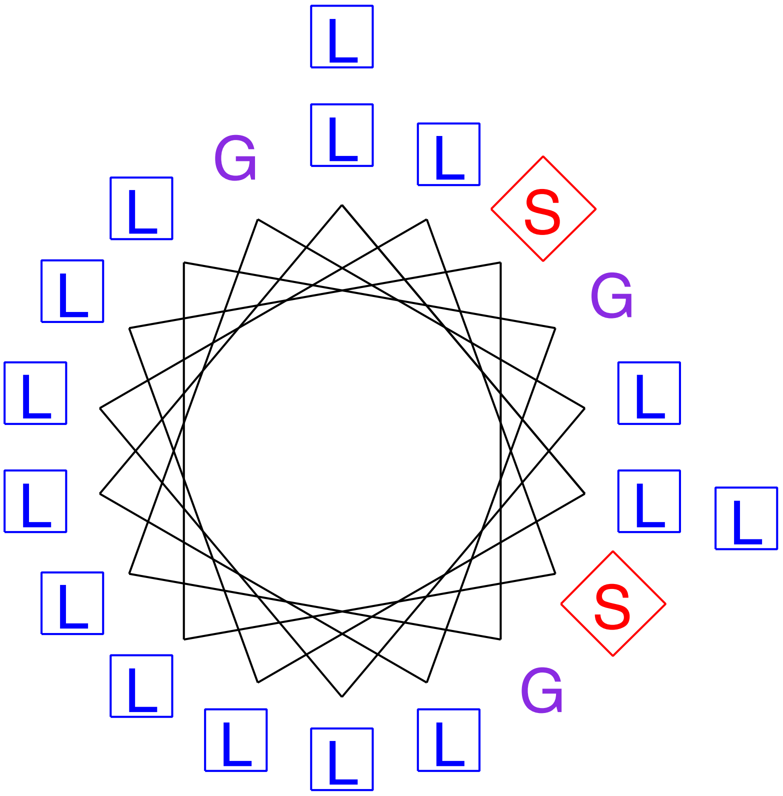

Fig. S2. Helical wheel representation showing mild amphipathicity of each transmembrane helix of REAMP. Plotted with EMBOSS Pepwheel at http://emboss.bioinformatics.nl/cgi-bin/emboss/pepwheel

SUPPLEMENTARY FIGURE S3

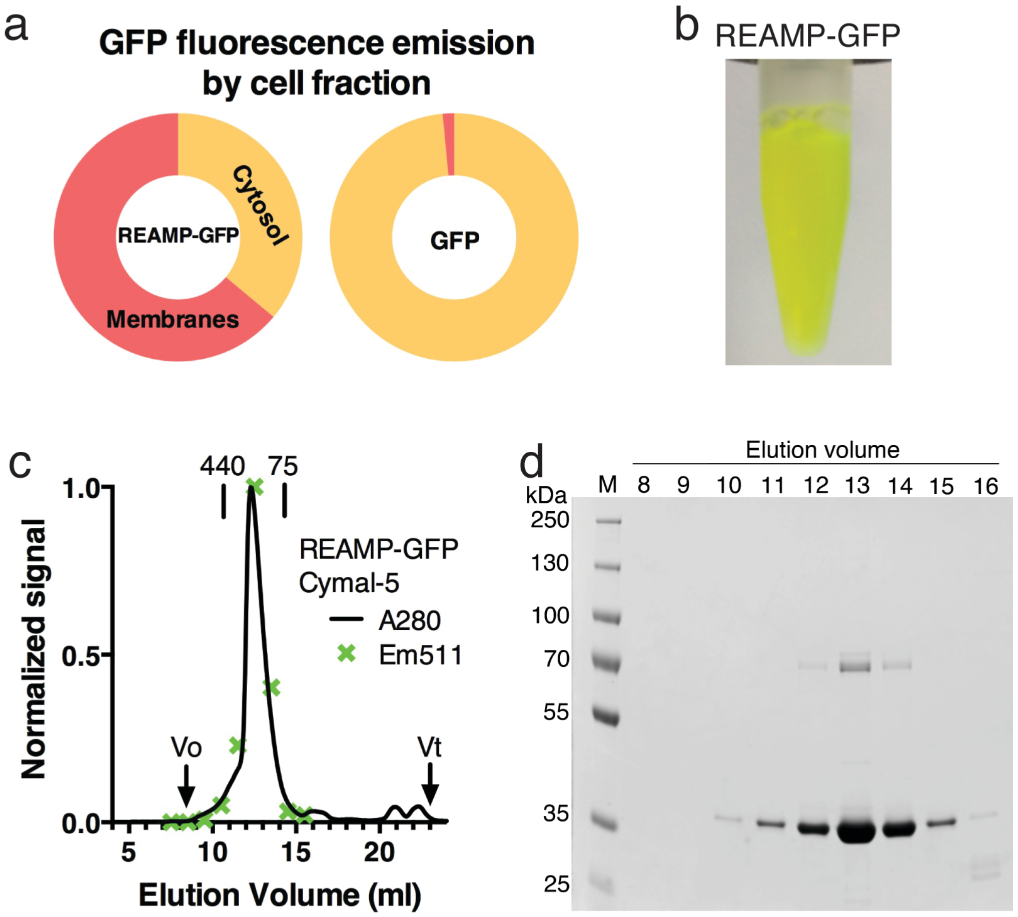

Fig. S3: Purification of REAMP-sfGFP-His_{10} (REAMP-GFP). (**a**) The fusion construct localizes sfGFP to the cell membrane, in contrast to control cells expressing sfGFP only. (**b**) Purified REAMP-GFP is brightly fluorescent. (**c**) REAMP-GFP is a single peak on size-exclusion chromatography and the protein absorbance signal (*A280*) corresponds to the GFP fluorescence emission collected off-line (*Em511*). (**d**) SDS-PAGE analysis after sample boiling shows that the SEC peak comprises a single species that runs close to the theoretical molecular weight for REAMP-GFP of 40.8 kDa. The minor bands at higher molecular weight are REAMP-GFP dimers that are largely, but not entirely, disrupted by sample boiling. The multiple bands represent different SDS-binding isoforms. Visualisation by Coomassie staining, gel is 10% acrylamide.

SUPPLEMENTARY FIGURE S4

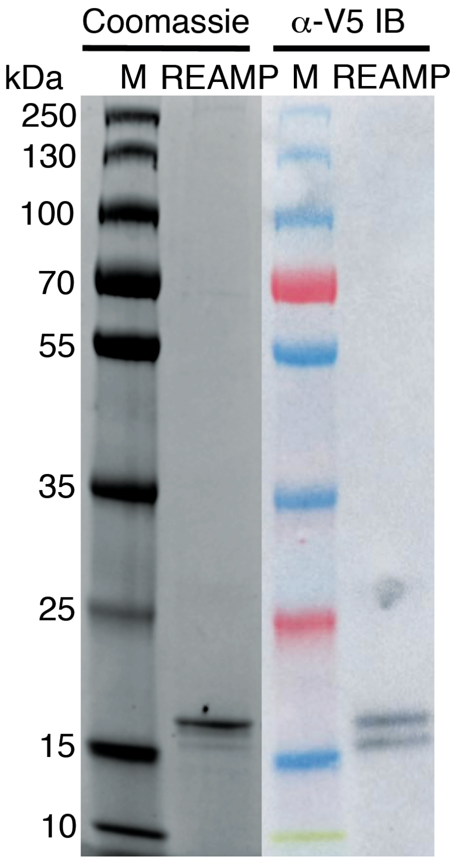

Fig. S4. Purification of REAMP with C-terminal His-tag via IMAC. The purified protein resolves as multiple bands on coomassie-stained SDS-PAGE and a doublet in Western blotting *(α-V5 IB)*, suggesting different modes of SDS binding. The doublet was not resolved by boiling the sample. Amino acid analysis (Fig. S5) confirms that only a single protein species is present.

SUPPLEMENTARY FIGURE S5

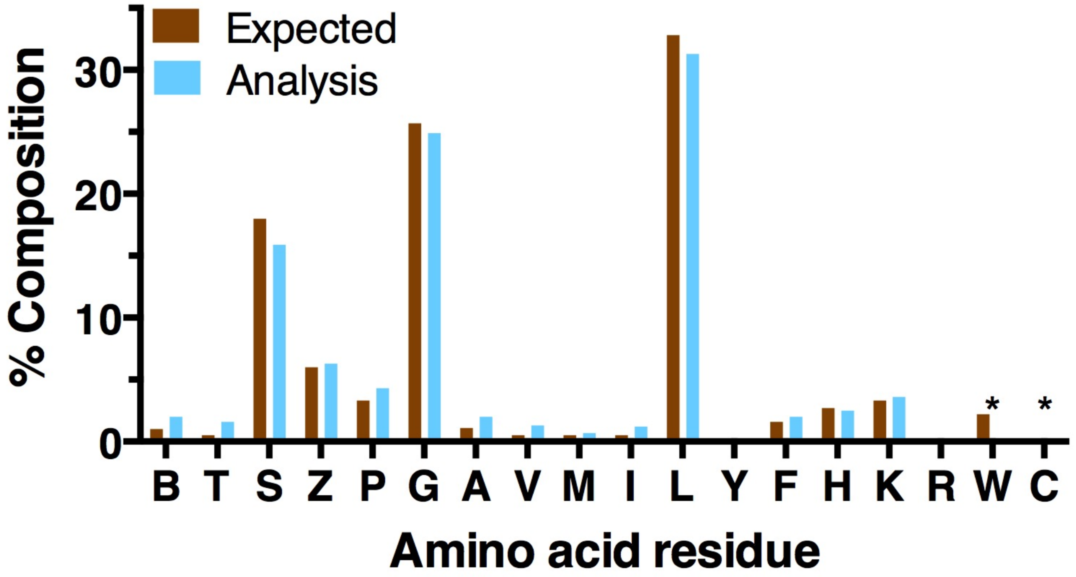

Fig. S5. Amino acid analysis of purified REAMP-Strep3 after acid hydrolysis. The experimental data (*Analysis*) matches the theoretical composition (*Expected*). The one-letter amino acid code is used. W and C cannot be accurately quantified by this method.

SUPPLEMENTARY FIGURE S6

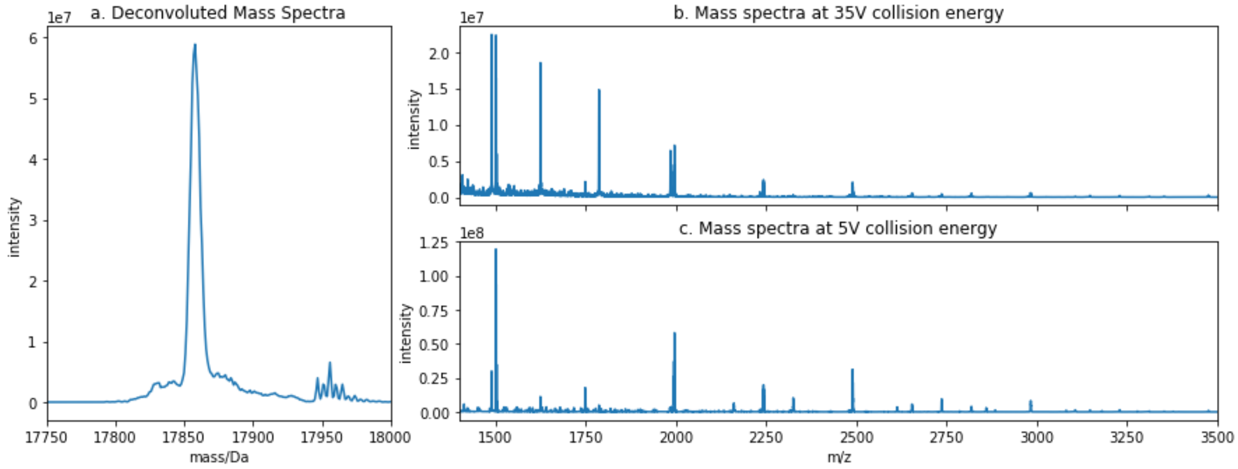

Fig. S6. Nanoelectrospray MS only identifies REAMP-Strep3 monomers (17.8 kDa) even at the lowest collision energies tested.

SUPPLEMENTARY FIGURE S7.

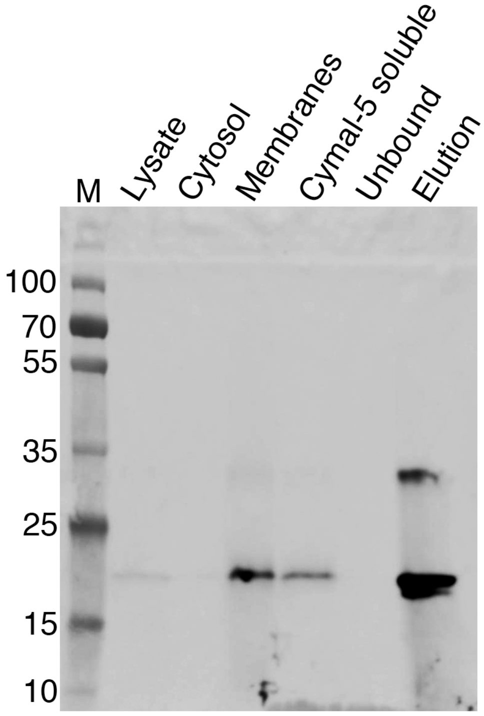

Figure S7. Uncropped western blot of REAMP-Strep3 purification. The cropped version appears in the lower panel of Fig. 2a.

SUPPLEMENTARY FIGURE S8

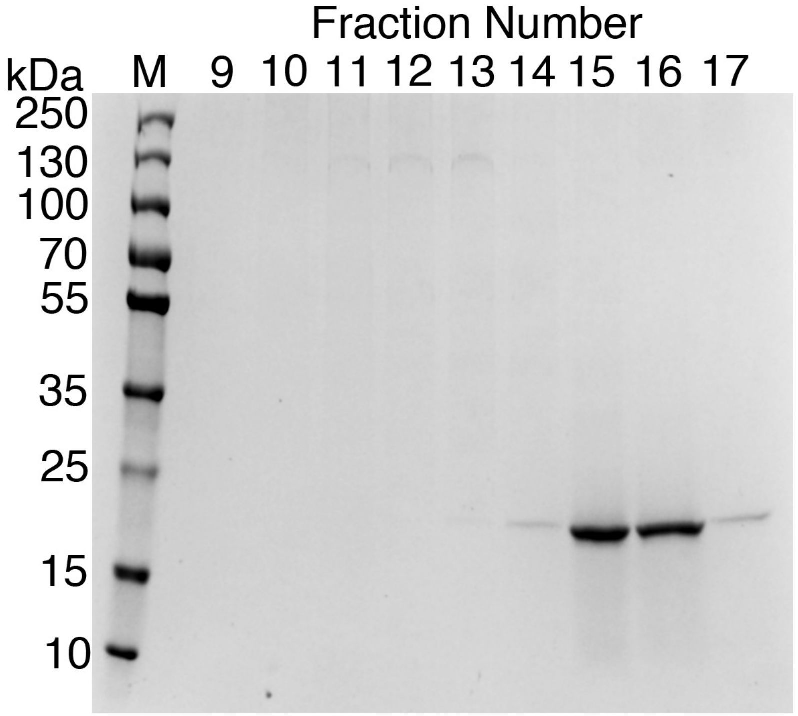

Fig. S8. Uncropped Coomassie-stained SDS-PAGE gel of fractions from size-exclusion chromatography of REAMP. The cropped version of this figure appears in Fig. 2b.

SUPPLEMENTARY FIGURE S9

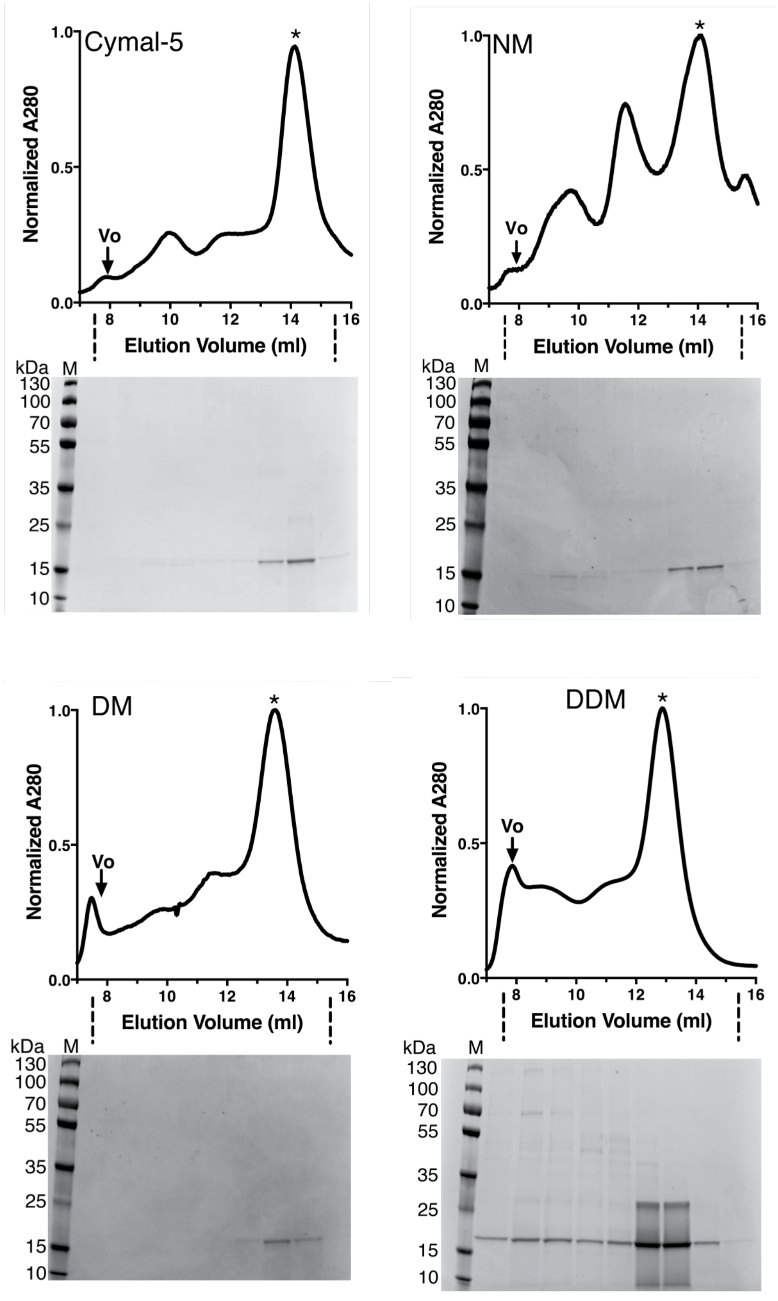

Fig. S9. Purification of REAMP in four different maltoside detergents as described in the text. In each case the major peak marked with a (*) is used for the apparent molecular mass of the protein-detergent complex (MW_{app} PDC) in Fig. 2c.

SUPPLEMENTARY FIGURE S10

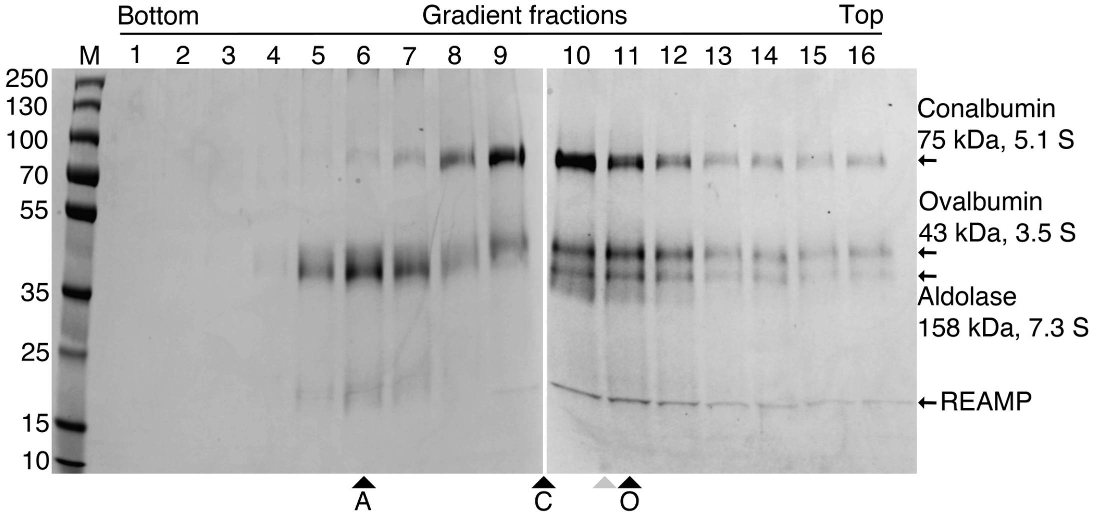

Fig. S10. Sucrose gradient ultracentrifugation of REAMP. Triangles below the gel represent the gradient position of Aldolase (A), Conalbumin (C), and Ovalbumin (O). The grey triangle shows the gradient position of REAMP. Aldolase is a tetramer of 158 kDa during gradient centrifugation but dissociates to a monomer (40 kDa) on SDS-PAGE.

SUPPLEMENTARY FIGURE S11

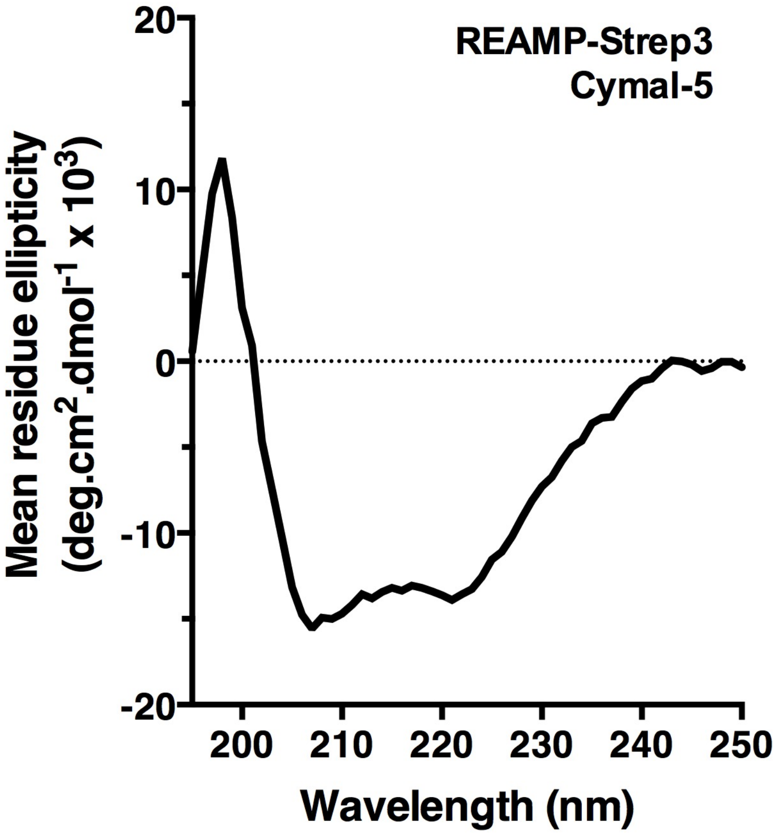

Fig. S11. Circular dichroism spectroscopy of triple *Strep*II-tagged REAMP in Cymal-5 confirms the expected α-helical structure.

SUPPLEMENTARY FIGURE S12

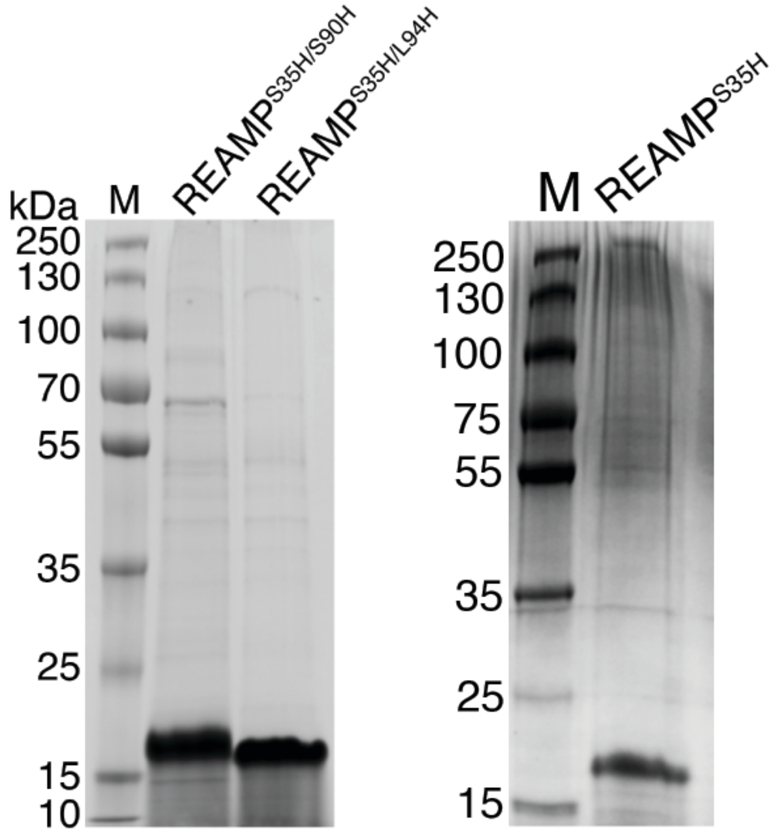

Fig. S12. Coomassie-stained SDS-PAGE gels showing the His-tag purification of bis-His and mono-His variants as shown.

SUPPLEMENTARY FIGURE S13

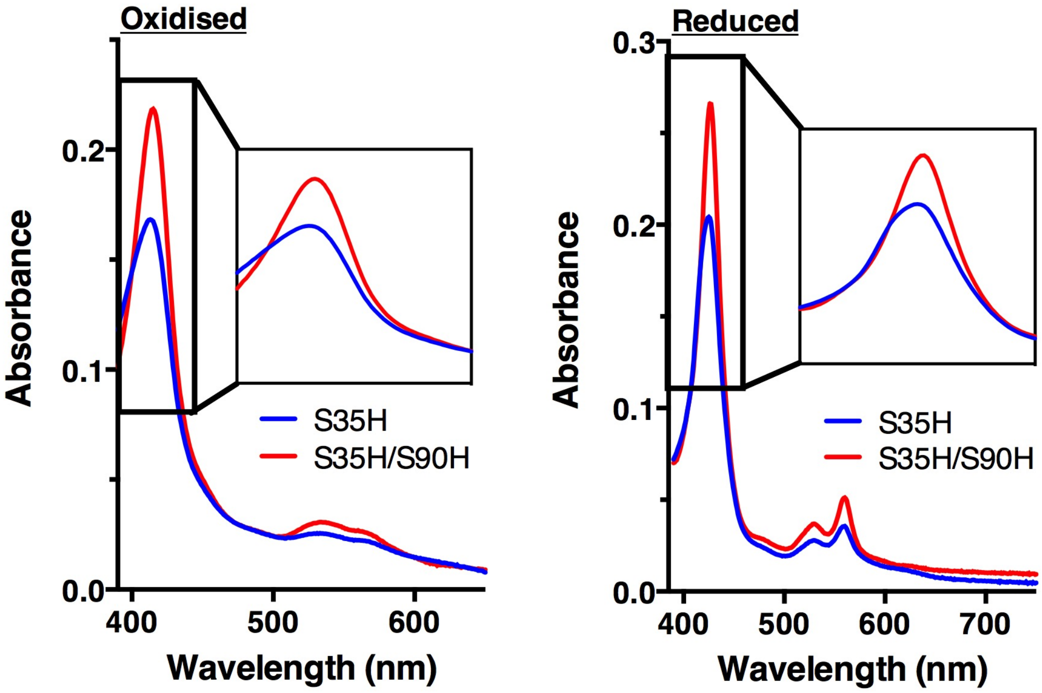

Fig. S13. Spectra of oxidized and dithionite-reduced heme co-ordinated by the mono-His and bis-His mutants of REAMP as shown.

SUPPLEMENTARY FIGURE S14

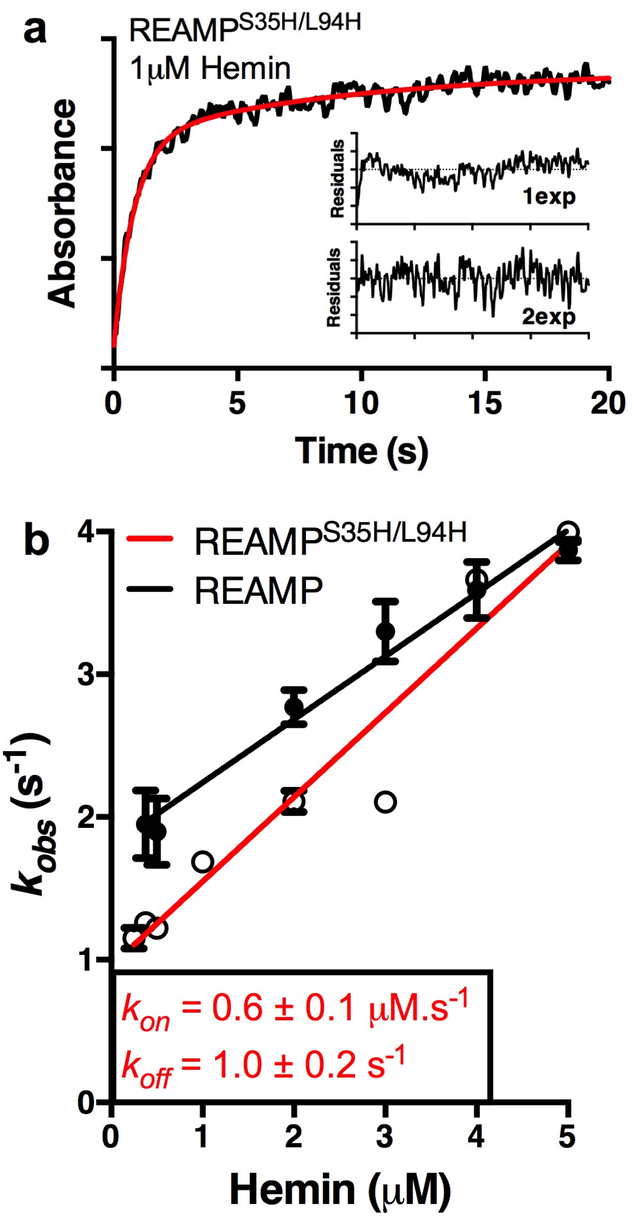

Fig S14. (a) Kinetics of heme binding to REAMP and a bis-His variant. Substrate binding kinetics were best fit to the sum of two exponentials, with the observed rate constant (k_{obs}) of the faster component being linear with heme concentration. (b) Linear extrapolation of the fast component yields k_{on} and k_{off} for REAMP$^{S35H/L94H}$ as shown. For REAMP the comparable on-rate (k_{on}) and off-rate (k_{off}) were 0.4 ± 0.03 μM.s^{-1} and 1.8 ± 0.1 s^{-1} respectively.

SUPPLEMENTARY FIGURE 15

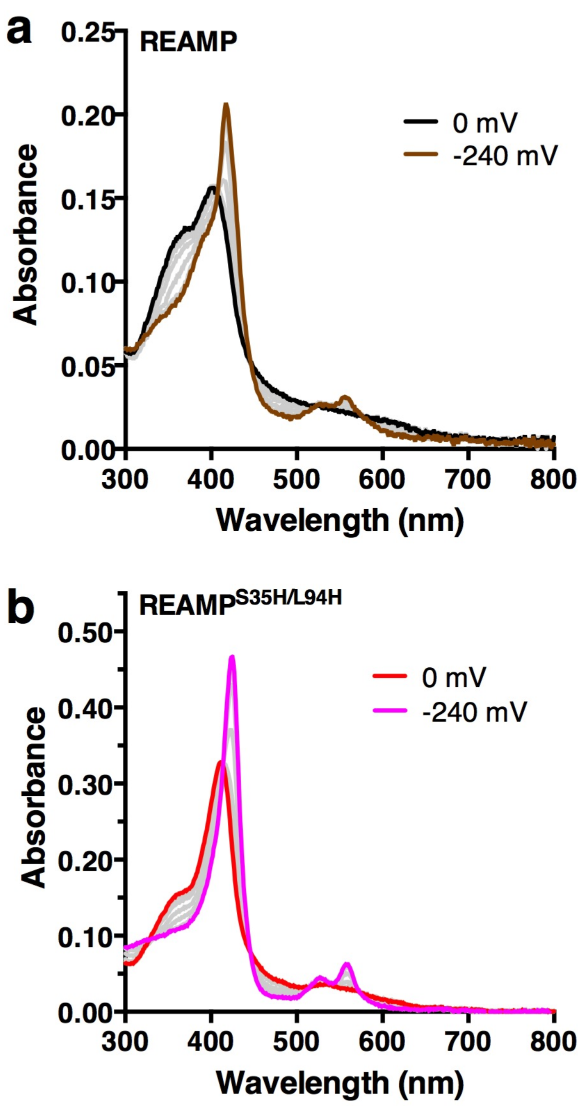

Fig. S15. Redox potentiometry. The absorption spectra of heme incubated with (**a**) REAMP and (**b**) REAMP$^{S35H/L94H}$ change predictably upon electrochemical reduction to -240 mV.

SUPPLEMENTARY REFERENCES

1 Krogh, A., Larsson, B., von Heijne, G. & Sonnhammer, E. L. L. Predicting transmembrane protein topology with a hidden markov model: application to complete genomes. *Journal of molecular biology* **305**, 567-580 (2001).

2 Tusnády, G. & Simon, I. The HMMTOP transmembrane topology prediction server. *Bioinformatics* **17**, 849-850 (2001).

3 Reynolds, S. M., Käll, L., Riffle, M. E., Bilmes, J. A. & Noble, W. S. Transmembrane topology and signal peptide prediction using dynamic bayesian networks. *PLoS Comput Biol* **4**, e1000213 (2008).

4 Peters, C., Tsirigos, K. D., Shu, N. & Elofsson, A. Improved topology predictions using the first and last hydrophobic helix rule. *Bioinformatics* **32**, 1158-1162 (2015).

5 Tsirigos, K. D., Peters, C., Shu, N., Käll, L. & Elofsson, A. The TOPCONS web server for combined membrane protein topology and signal peptide prediction. *Nucleic Acids Res* **43**, W401-W407 (2015).
